# National Nutrition Communication Campaign in Indonesia: a cross-sectional study of factors associated with exposure

**DOI:** 10.1186/s13690-021-00697-y

**Published:** 2021-10-09

**Authors:** Leiema Hunt, Abigail Norton, Chantel Daines, Evie Friedbaum, Danica Topham, Ryan Moffat, Scott Torres, Mary Linehan, Hafiza Jusril, Cougar Hall, Benjamin Crookston, Josh West

**Affiliations:** 1 Department of Public Health, Brigham Young University, Provo, UT USA; 2 College of Dental Medicine, Roseman University, South Jordan, UT USA; 3 Senior Manager Neglected Tropical Diseases, RTI International, Washington, D.C., Senior Program Director, IMA World Health, Washington, D.C., USA; 4 Infectious Disease Team Lead, USAID, Jakarta, Indonesia; 5 Associate Researcher, Reconstra, Jakarta, Indonesia

**Keywords:** National Communications Campaign, Stunting, Interpersonal communications, Health behaviors, Indonesia

## Abstract

**Background:**

Reducing childhood stunting continues to be a priority in Indonesia. In 2015, the National Nutrition Communication Campaign (NNCC) implemented mass media and interpersonal communication (IPC) interventions to disseminate stunting-related information. Whereas other studies of the NNCC’s impact on attitudes and behaviors are currently underway, the purpose of this study was to better understand the factors associated with exposure to the media and IPC components of the NNCC.

**Methods:**

A cross-sectional survey was conducted following the NNCC media and IPC campaigns in rural Indonesia. The final study sample included 1734 mothers. Survey data was collected from each participant by trained interviewers using an electronic tablet. Responses relating to demographic and socioeconomic factors, use of social media and WhatsApp, and electronic device ownership were analyzed. Logistic regression analyses, using SAS version 9.4, were conducted to evaluate the relationship between technology-related items and exposure to both the media and the IPC interventions.

**Results:**

Owning an internet device (OR = 1.643, CI = 1.237–2.183, *p* < 0.001), accessing social media (OR = 1.81, CI = 1.32–2.49, *p <* 0.001), using a device to access health information (OR = 2.068, CI = 1.469–2.911, *p* < 0.0001), and accessing WhatsApp (OR = 1.663, CI = 1.175–2.355, *p* < 0.05) were positively related to exposure to NNCC messages meant to change behavior to decrease stunting. In separate analyses, owning an internet device (OR = 0.609, CI = 0.459–0.81, *p* < 0.001) accessing social media (OR = 0.626, CI = 0.459–0.854, *p* < 0.05), using a device to access health information (OR = 0.528, CI = 0.377–0.740, *p <* 0.001), and accessing WhatsApp (OR = 0.688, CI = 0.489–0.968, *p <* 0.05) were negatively related to IPC exposure. Mothers with access to internet-accessible devices were more likely to be exposed to the media campaign component to decrease stunting while mothers without access to internet-accessible devices were more likely to be exposed through IPC.

**Conclusions:**

Mothers who owned devices that could access the internet were more likely to have been exposed to the media campaign component to decrease stunting by behavior change but were less likely to participate in IPC activities. The opposite was true for mothers who did not have access to internet-accessible devices. These findings may be used to inform future community health efforts in rural Indonesia and similar regions that may be considering the use of both mass media and interpersonal interventions to influence health behaviors in order to decrease stunting.

## Background

Stunted growth occurs when a child’s height-for-age Z-score is more than two standard deviations below the World Health Organization (WHO) growth standards median [1]. It is the result of inadequate nutrition and chronic infections and can increase the risk of delayed cognitive development, reduced intellectual capacity, poor school performance, and increased morbidity and mortality [2]. In the first 1000 days of life, from conception until the age of two, proper nutrition for both mother and child is essential to reduce the risk of stunting.

Indonesia ranks 5th in the world for stunting prevalence with approximately 9.2 million (37%) children stunted [2, 3]. At 40%, stunting rates among rural Indonesians are higher than the national average of 31% [3]. Among the poorest Indonesians, stunting affects 43% of children while among the wealthiest fifth of the population, just 24% of children are affected [3].

The National Nutrition Communication Campaign (NNCC) aimed to address stunting by influencing the beliefs and behaviors of mothers and other caregivers in families with children under the age of two. As part of the NNCC, both a media campaign and an interpersonal communication (IPC) campaign were developed and deployed. The media campaign was implemented nationally and consisted of TV commercials, talk shows, documentaries, and the social media platforms of Facebook, Twitter, and Instagram. At a more local level (e.g., district and village), the IPC campaign consisted of one-on-one counseling classes for mothers. The NNCC was based on the Theory of Planned Behavior (TPB), which postulates that behavioral intentions are the primary determinants of behavior and are predicted by an individual’s attitudes and beliefs about the behavior, social and subjective norms related to the behavior, and perceived behavioral control or self-efficacy for the behavior [4]. NNCC media and IPC interventions were designed to improve nutritional outcomes and prevent childhood stunting by targeting each of these theoretical constructs. For example, key messages were transmitted through an opinion leader (subjective norm) in an effort to impact normative beliefs and attitudes supportive of desired behavior intentions and change [5]. The IPC component was intended to develop skills to increase perceived behavioral control and self-efficacy predictive of behavior change. Theoretical approaches of this nature have been effective for modifying health-related behaviors, including efforts to promote complimentary feeding [6–9].

Whereas the impact of media campaigns on health behaviors in rural, developing settings has been studied, the factors associated with the exposure to media campaigns in rural Indonesia are largely unknown [10]. The purpose of this study was to examine the factors associated with exposure to the media component and IPC component of the NNCC in order to provide information necessary to inform future programs how to influence health behaviors and impact stunting in Indonesia. On account of the solid theoretical underpinnings for the NNCC, there is reason to believe that exposure to the campaign would result in an impact on health behaviors related to nutrition. An analysis of this nature is beyond the scope of the current study.

## Methods

### Study design, setting and sampling

Data for this study came from a cross-sectional survey conducted following the NNCC media and IPC campaigns in Indonesia. Data were collected in rural districts (Banyuasin, Kubu Raya, and Katingan) located in three provinces (South Sumatera, West Kalimantan, and Central Kalimantan). One district was randomly selected from each of the three provinces. Within districts, three-stage cluster sampling was applied to villages as cluster units utilizing a random sample method. Based on power analyses, an initial sample of 600 mothers was targeted for participation in the follow-up survey in each district, which would have resulted in a total sample of 1800 (600 per district × 3 districts). On account of some instances of missing data and, the final study sample for analyses included 1734 mothers. Although fathers were not targeted in this study, because of the possibility that they had influence in decisions made by the mother, their presence was noted.

### Procedures

Ethical approval was acquired from the Ethical Research Committee by the Faculty of Public Health, University of Indonesia. Signed informed consent was sought from each participant prior to the interview and the participation of all subjects was on a voluntary basis. Survey data was collected from each participant using an electronic tablet by experienced interviewers and field coordinators who were trained extensively. Each interviewer was responsible for interviewing six respondents per day and then report to field coordinators, who then verified the responses and uploaded survey data daily. A data manager also checked the data and corrected any errors. Data cleaning was done prior to analysis. Each variable was labeled, and all data was transferred to the SAS statistical data software for analysis.

### Measures

Demographic information was collected including the mother’s age, total household income, and level of education. A series of technology ownership and access-related indicators were also measured. First, respondents were asked to simply report (yes/no) about ownership of several common electronic devices, including mobile phones, smartphones, tablets, and laptops/computers. A new variable was created that reflected device ownership and it included all respondents that reported ownership of at least one of these devices. A second indicator was created to reflect ownership of internet-accessible devices, only. This included smartphones, tablets, and laptops/computers. This indicator was created because of the media component’s emphasis on web-based distribution channels. Lastly, respondents were asked to report (yes/no) about whether or not they had used their device to access social media and WhatsApp. Social media included having accessed Facebook, Twitter, or Instagram. WhatsApp was included as it is a free communication platform and widely used in Indonesia. Lastly, respondents were asked to report whether or not they had ever used a device to access health information on the internet (yes/no).

To ascertain media exposure, interviewers showed respondents a brief video clip of the TV commercials or an image of the print media or social media page and asked if the they had seen the particular media. Exposure was confirmed by asking the respondent to describe the theme or message in the commercial, print media or social media. Respondents were considered to be exposed to the media component with a valid confirmation of exposure to any of the various platforms. Exposure to the IPC component was determined by asking respondents if they had participated in mothers’ classes or support groups for mothers of children below the age of 2 who meet regularly to share experience, discuss, and give support for mother and child’s health primarily related to pregnancy, breastfeeding, and nutrition which was facilitated by the *Posyandu* (an Indonesian community health outpost). If respondents said yes, they were asked to provide a description of the topics of the meetings. Respondents were considered to be exposed to the IPC component following confirmation of exposure to any of the IPC meetings.

### Statistical analysis

Descriptive statistics were calculated for demographic variables. All analyses were conducted using SAS version 9.4. Logistic regression analyses were conducted to evaluate the relationship between technology-related items and exposure to both the media and the IPC interventions, separately. Each model controlled for the mother’s age, level of education and total household income.

## Results

Table 1 presents demographic information of study participants. The average age of respondents was 28.9 years, and most had completed some form of primary education. More than half of the sample reported owning a mobile phone (Table 2), with just over one-third of the sample owning a smartphone. Considering all devices, over two-thirds (67.47%) reported ownership of an electronic device and 31.72% owned a device that was internet-ready. Most of the sample reported exposure to the media component (74.74%), while just over one-fourth were exposed to IPC (26.47%) (Table 3).

**Table 1:** Demographic characteristics of the participants in the cross-section survey, *n* = 1734

*Total annual household income*^a^	Mean	SD
	2,193,594.18	1,944,608.71
*Age*		
Mother	28.94	6.25
Father	32.96	7.11
	**N**	%
*Mother’s Education*		
None	97	5.59
Primary	670	38.64
Junior	423	24.39
Tertiary	434	25.03
College/University	110	6.34
*District*		
Banyuasin	605	34.89
Kubu Raya	598	34.49
Katingan	531	30.62

^a^Indonesian Rupiah (IDR)

**Table 2:** Device ownership of participants in survey, *n* = 1734

***Devices***	N	(%)
Mobile phone	999	57.61
Smartphone	619	35.70
Tablet	79	4.56
Laptop/Computer	75	4.33
Any device^a^	1170	67.47
Internet device^b^	550	31.72

^a^Owned a mobile phone, smartphone, tablet, or laptop/computer.

^b^Owned an internet-ready device, smartphone, tablet, or laptop/computer

**Table 3:** Exposure to NNCC components of participants in survey, *n* = 1734

	N	(%)
Interpersonal Communication (IPC)	459	26.47
Media	1296	74.74

Respondents could report access to both IPC and Media.

### Differences between IPC and media groups

Table 4 shows the frequency of respondent’s use of electronic, internet-ready devices. Less than a quarter of respondents used their devices to access social media, including Facebook, Twitter, or Instagram, just under 20% used their device to access health information, and 17.76% used their device to access WhatsApp. In every category, respondents that reported exposure to the media component also reported higher use of electronic devices.

**Table 4:** Use of electronic devices by participants with respect to IPC and Media, *n* = 1734

	Total N (%)	IPC N (N%)	Media N (%)
Social media access	395 (22.78)	82 (20.76)	325 (82.28)
Health technology	342 (19.72)	64 (18.71)	284 (83.04)
WhatsApp access	308 (17.76)	65 (21.10)	251 (81.49)

### Technology access and exposure to digital media campaign

In both the unadjusted and adjusted models, owning an internet device (OR = 1.64, 95% CI = 1.24–2.18) accessing social media (OR = 1.81, 95% CI = 1.32–2.49), using a device to access health information (OR = 2.07, 95% CI = 1.47–2.91), and accessing WhatsApp (OR = 1.66, 95% CI = 1.18–2.36) were all significantly and positively associated with exposure to the media component of the campaign as shown in Table 5.

**Table 5:** Logistic Regression of Factors associated with exposure to the media component of NNCC, *n* = 1296

***Dependent variables***	Unadjusted OR (95% CI)	Adjusted OR (95% CI)
Own device	0.98 (0.74–1.34)	0.89 (0.61–1.30)
Internet device	1.53 (1.23–1.96)**	1.64 (1.24–2.18)**
Social media access	1.76 (1.32–2.34)**	1.81 (1.32–2.49)**
Health technology	1.84 (1.35–2.50)***	2.07 (1.47–2.91)***
WhatsApp access	1.61 (1.18–2.19)*	1.66 (1.18–2.36)*

* *p <* 0.05; ** *p <* 0.001; *** *p* < 0.0001.

All adjusted models include maternal age, maternal education level, and total household income

### Technology access and exposure to IPC

In both the unadjusted and adjusted models, owning an internet device (OR = 0.61, 95% CI = 0.46–0.81) accessing social media (OR = 0.63, 95% CI = 0.46–0.85), using a device to access health information (OR = 0.53, 95% CI = 0.38–0.74), and accessing WhatsApp (OR = 0.69, 95% CI = 0.49–0.97) were all significantly and negatively associated with exposure to the IPC component as shown in Table 6*.*

**Table 6:** Logistic Regression of Factors associated with exposure to the IPC component of NNCC, *n* = 459

***Dependent variables***	Unadjusted OR (95% CI)	Adjusted OR (95% CI)
Own device	1.08 (0.77–1.51)	1.22 (0.83–1.79)
Internet device	0.66 (0.52–0.83)**	0.61 (0.46–0.81)**
Social media access	0.67 (0.51–0.88)*	0.63 (0.46–0.85)*
Health technology	0.58 (0.43–0.78)**	0.53 (0.38–0.74)**
WhatsApp access	0.70 (0.52–.094)*	0.69 (0.489–0.97)*

* *p <* 0.05; ** *p <* 0.001.

All adjusted models include maternal age, maternal education level, and total household income

## Discussion

This study aimed to identify the factors associated with exposure to the mass media and IPC components of the NNCC. Mothers who owned an internet accessible device and used it to access health information were more likely to report exposure to the NNCC media campaign and less likely to be exposed to IPC, which was offered at local, community health outposts (*Posyandu*). The finding that the correlates for exposure to the mass media and IPC components were in opposite directions is interesting, as it is plausible that media exposure would foster an interest in or use of the available IPC information. Several previous studies have shown that when media is combined with IPC interventions, norms (influenced by the media) and skills (influenced by IPC) were more likely to promote a change in behaviors [9, 11]. IPC in coordination with mass media can act as a moderator and mediator of media effects and can also be a functional agent for behavior change [11–13]. At least one mechanism for this process could include a situation where an observer is exposed to mass media in rather impersonal tones (e.g., a brief television segment) that has an impact on knowledge acquisition, especially with repeated exposures. Then, from within a more comfortable or interpersonal network and from those that have the technical skills, the message is reinforced, which is likely to have a more profound impact on attitudes, beliefs and perceptions [14]. The possible interplay between the mass media and IPC interventions was beyond the scope of the current study, but future research could identify ways to improve the overall impact of interventions prioritizing both platforms.

A high number of study participants reported exposure to the NNCC media campaign. This is encouraging and comparable to other studies of large health-related campaigns in developing settings [15]. Indeed, the purpose of mass media campaigns is to expose high portions of large populations, ostensibly with the intent of changing behavior [16]. Under the right conditions, media campaigns can help to change knowledge, social norms and behaviors in positive ways [16–18]. High exposure to the media campaign may be somewhat expected based on rates of social media usage and exposure. Indonesia ranks third in the world for the number of Facebook users with about 123 million users, a contextual factor that could explain, at least in part, the comparatively high rate of reported media exposure [19]. NNCC’s media campaign was the first stunting-specific campaign in Indonesia to invest in social media and it has become a popular platform to share information and connect with groups that have the same concerns [20, 21].

Compared to the media component, a much smaller proportion of respondents reported exposure to IPC. In some respects, this is understandable because the level of effort required to participate in IPC was comparatively high. IPC was delivered at local health posts, which necessitated travel and any attending time and financial costs. The findings that respondents exposed to IPC were significantly less likely to engage with technology might be the result of a preference for face-to-face communication. Nevertheless, both media and IPC can be important. A focus for future practice may be to strengthen participation in IPC for individuals that do not have access to or prefer not to use communication technologies. This might include things like using incentives [12]. Insomuch as ownership and use of the various technologies correlated negatively with IPC exposure, it may be useful for future interventions in rural settings to continue to support and promote more traditional approaches for delivering interventions.

The results from this study should be considered in the context of its limitations. First, this study did not use an asset index to measure poverty, which is generally regarded as a more accurate indicator of wealth in developing settings. Since the indicators necessary to construct such an index were not included in the study survey, a measure of total household income was used instead. Second, the survey did not include measures of attitudes related to either of the two platforms. Favorable views toward either could potentially impact exposure. And lastly, due to high cost of data collection, the sample size of those exposed to IPC was comparatively small. It was still adequately powered for the analyses, but an assessment of additional variables that might be related to exposure would require a larger sample.

## Conclusions

This study, conducted in three different rural areas of Indonesia, expands current understanding and knowledge of the factors associated with mass media or IPC components. These results may be useful for future interventions in rural and developing settings, especially as digital communications mediums become more ubiquitous.

Practice Points:

- Stunting is impaired growth and development in children because of poor nutrition and psychosocial stimulation, and repeated infection; Indonesia has a high stunting prevalence.
- National Nutrition Communication Campaign disseminated information about the prevention and control of stunting using interpersonal communication (IPC) and mass media.
- Mothers who owned devices that could access the internet were more likely to have been exposed to media campaign component.
- Mothers who owned devices that could access the internet were less likely to participate in IPC related activities.
- Results suggest understanding and supporting participation in IPC for individuals that do not have access to or prefer not to use communication technologies.

## Data Availability

The datasets generated and/or analyzed during the current study are not publicly available due to proprietary ownership by IMA World Health but are available from the corresponding author on reasonable request.

## Abbreviations

IPC: Interpersonal communication
NNCC: National Nutrition Communication Campaign
TPB: Theory of Planned Behavior
WHO: World Health Organization
